# Computer Simulation of Partial Discharges in Voids inside Epoxy Resins Using Three-Capacitance and Analytical Models

**DOI:** 10.3390/polym12010077

**Published:** 2020-01-02

**Authors:** Johnatan M. Rodríguez-Serna, Ricardo Albarracín-Sánchez, Ming Dong, Ming Ren

**Affiliations:** 1 Department of Electrical and Electronic Engineering, Automatic Control, and Applied Physics, School of Industrial Design and Engineering (ETSIDI), Universidad Politécnica de Madrid (UPM), Ronda de Valencia 3, 28012 Madrid, Spain; 2 State Key Laboratory of Electrical Insulation for Power Equipment, Xi’an Jiaotong University, Xi’an 710049, China; dongming@mail.xjtu.edu.cn (M.D.); renming@mail.xjtu.edu.cn (M.R.)

**Keywords:** partial discharges, epoxy resin, three-capacitance model, induced-charge concept, insulation ageing, condition monitoring

## Abstract

Epoxy resin is one of the most common polymers used as part of the insulation system in key electrical assets such as power transformers and hydrogenerators. Thus, it is necessary to know their main characteristics and to evaluate their condition when subjected to High Voltage (HV). A brief review of epoxy resins’ applications as insulating materials is made, their main characteristics as insulating media are given, the improvements with nano-fillers are summarized and the main electric properties required for Partial Discharges (PD) modelling are listed. In addition, the theoretical background and state-of-the-art of the three-capacitance and analytical models for simulating PD in solid dielectrics, such as epoxy resins, are reviewed in detail. Besides, their main advantages and disadvantages are presented, some critical arguments to the modelling procedure and assumptions are made and some improvements are proposed, taking into account conclusions made from other authors using models related to the PD development process. Finally, a case study was simulated using a modified three-capacitance model and the analytical model. The PD rate, q-φ-n diagrams and the minimum, mean and maximum PD electric charge are compared with measurements reported in the literature. Simulation results are in reasonable agreement with measured values. Capacitance models can be implemented in general purpose electric circuit simulation packages; however, its simulation is computationally expensive. Additional to this, although the modified three-capacitance model is not as accurate as finite elements or analytical models, results are also in agreement with real data.

## 1. Introduction

Modern societies mostly demand a constant and reliable supply of energy which indeed requires an increased availability of equipment in power systems [1]. Solid dielectric materials are fundamental for the adequate operation of electrical equipment; they are used as insulating materials in applications ranging from electronics to high voltage equipment [2]. Polymeric materials appear as a novel alternative to traditional insulating materials because they have many advantages such as, elasticity, cost of manufacturing, resistance to chemicals and thermal stability [3]. In addition, characteristics such as, breakdown voltages, relative permittivity and dissipation factor can be modified using additives and nanofillers [4]. Epoxy resin is one of the most successful polymers for electrical insulation systems in power equipment; it has good mechanical and electrical characteristics, has good adhesion and minimal shrinkage during curing. In addition, volatile by-products are not produced during the curing process [5].

When electric devices and equipment are connected to a power grid, usually they cannot be disconnected for commissioning in order to evaluate the real operating conditions of the insulation system. However, power equipment, such as power transformers and generators, are expensive and availability and adequate operating conditions are essential for stability and reliability of power systems. The most frequent sources of failure in power transformers and hydrogenerators are related to weaknesses in their insulation systems [6]. Additionally, PD in cavities are one of the main causes of dielectric breakdown in solid insulators and are also symptoms of ageing in high-voltage (HV) equipment [7,8,9]; indeed, in hydrogenerators, 22% of failures are due to internal partial discharges [10]. According to the IEC 60270 standard [11], PD are localized electrical discharges that only partially bridges the insulation between conductors and which can or cannot occur adjacent to a conductor. They are very-fast-transient phenomena that appear due to a high-field enhancement that exceeds the local dielectric strength, for instance, inside cavities.

The modelling and simulation of PD allow us to analyse the process from an incipient state to breakdown and to understand the dielectric ageing process [12] as well as its relationship with factors and variables such as temperature, voltage amplitude and frequency [13]. In addition, PD simulation can complement measurement systems in order to improve diagnosis procedures [14].

In order to model the PD activity in voids inside epoxy resins, there currently are models that can be classified as the following [15,16,17]:

- Three-capacitance or “abc” model.
- Analytical model.
- Finite-Element-Algorithms (FEA) model.

As expected, FEA models are more accurate than others, allowing us to consider the non-uniform distribution of the electric field, the real PD charge distribution and the multi-stress conditions for PD phenomena [18]. In addition, variables such as currents, real and induced charge and voltages can be calculated dynamically and correlated with experimental data [19]. FEA models have been recently used for modelling the PD in gas-filled cavities as filamentary dielectric barrier discharges where fluid equations are used to simulate the PD development process, considering the ionization process, charge drift, diffusion, recombination [20], the plasma chemistry and boundary phenomena [21]. Those plasma models allow to investigate PD inside voids for different stages of discharge activity and show good agreement when compared with measured and simulated values [22]. However, using FEA models requires high-computational consumption, which hinders the simulation of multiple PD, as for example, for simulating phase-resolved partial discharge (PRPD) patterns or q-φ-n diagrams for hundreds or thousands of periods of applied voltages at a power frequency. On the other hand, considering basic variables such as PD rate and PD charge, analytical and FEA models present very similar results when compared with experimental results [15].

Analytical and three-capacitance models can be useful when small void sizes are considered; the charge distribution along the void surface is not required and short-simulation time is necessary as in the case of ageing process analysis [23].

In this paper, the main characteristics and dielectric properties of epoxy resins for applications as insulating material are presented. In addition, a comparison between analytical and three-capacitance models is made in order to show its advantages for using them in academic and condition monitoring environments for PD modelling in epoxy resins. A case study was simulated using both models and results are presented to be compared with experimental values reported in the literature. It is organized as follows. First, a review of the epoxy resins characteristics and applications as insulating materials is presented in Section 2. Second, the state-of-the-art and the theoretical background of the three-capacitance and analytical models are presented in Section 3. Then, a case study and the simulation results are presented, respectively, in Section 4 and Section 5. Finally, some conclusions are drawn in Section 6.

## 2. Epoxy Resins Characteristics, Properties and Applications as Insulating Materials

Epoxy resins are cross-linked polymers produced by a polymerization reaction between a fluid prepolymer that form linear chains (base resin), and a hardener (curing agent) [24]. Their properties depend on the specific type of epoxy resin and curing agents used, Table 1 shows a summary of epoxy resins used in industrial applications and its characteristics [25].

As curing agent, anhydride and amine type are basic. The latter can be classified as aliphatic, aromatic, or cycloaliphatic. Anhydride agents have ideal characteristics for dielectrics [25].

During the preparation of epoxy resins, in order to obtain a highly cross-linked three-dimensional polymeric network, a curing process in which chemical reactions of epoxide groups and curing agents (hardener) is required [26]. The curing process can be done at room temperature or at elevated temperatures, higher than 100 °C [27]. Room temperature cured epoxy resins have a lower glass transition temperature (T_g_), higher flexibility, greater impact resistance, and greater electrical and thermal shock resistance. On the other hand, epoxy resins cured at greater temperatures have a higher T_g_, greater tensile strength, higher heat resistance, greater chemical resistance resins and excellent electrical properties [25].

In room temperature curing processes, the hardener is generally an amine (aliphatic, aromatic, or cycloaliphatic) such as diethylene triamine or triethylenetetramine, while for elevated temperature curing processes, a number of different curing agents could be utilized, including aromatic amines and acid anhydrides [3]. Curing processes can also be done by photoirradiation using catalytic curing agents such as benzylsulfonium, benzylpyridinium, benzylammonium, and phosphonium salts [25]. Table 2 shows a list of amine curing agents used in epoxy resins and its main characteristics [3].

Acid anhydrides have good solubility in resins and are used in castings and laminates applications. Table 3 shows a list of anhydride curing agents used in epoxy resins and its main characteristics [3].

The properties of epoxy resins depend on the conditions of preparation, and in order to attain the best mechanical and electrical properties, variables such as temperature, humidity and pressure have to be controlled [28], specially for avoiding void formation and minimizing residual mechanical stresses [5].

Temperature is critical in order to achieve the correct cross-linking density. The mechanism of epoxy resin curing is complex, and the adequate preparation must consider the kinetic dynamics under conditions different from those of the laboratory. The rate of polymerization increases with temperature [27]. At greater temperatures and/or heating rates, epoxy materials cure at about 100%, which is characterized by the glass transition temperature (T_g_). On the other hand, under isothermal conditions below T_g_, epoxy materials do not cure completely, the curing process stops below 100%, and the percentage diminishes as the temperature is reduced below T_g_ [29].

The curing process is affected by gelation and vitrification processes [30], which are related to the epoxy system mobility. At the beginning, the curing process rate is controlled by chemical reactions and at the gel point, the growing individual macromolecules become connected into a single network and the epoxy system cannot flow but it is able to achieve long-range motion of polymer chain segments. Additional crosslinking within the network leads to vitrification, the polymer system has not long-range mobility and the polymerization process stops. At this point, the rate of curing process is controlled by diffusion. There is a change in the curing process regime from kinetic to diffusion. In [31] the processes of gelation and vitrification were analyzed using Temperature Modulated Differential Scanning Calorimetry (TMDSC) and Dynamic Rheometry (DR) methods in a DGEBA—m-PDA epoxy compound. It was found that that the onset of the diffusion regime may be associated with gelation rather than vitrification.

The properties of the cured epoxy resin depend on the cross-linking state and the adequate cure must be evaluated. In [32], a dimensionless criterion is proposed for quantifying the curing state of a thermoset. The cure index is calculated as a function of temperature and the total heat values released during the cure reaction taken from nonisothermal Differential Scanning Calorimetry (DSC) thermograms. The advantage of this criterion is that information about chemistry of curing reactions is not needed. In addition, it is found that polymeric systems containing a reactive additive, experience an efficient cure because they allow to control the chemical kinetics during the early state of curing process, retarding the gelation and vitrification phenomena appearance.

The value of T_g_ is related to the degree of cure. Some studies, related to partial discharges in tree channels, have found that conductive trees appear in glassy epoxy resins and non-conductive trees in epoxy resins above their glass transition temperature [33], which can be a result of carbonized products, due to PD activity, condensing on the surface of glassy epoxy resins [34]. In this work, it is asssumed that epoxy resins are above its glass transition temperature, in its flexible state.

One of the main advantages of epoxy resins for insulation applications is that they are thermosetting resins that can be castable into rigid structures forming solid geometries in which the electric field distribution is uniform. On the other hand, they can be impregnable as fluids or powder into porous surfaces such as, enameled wires or mica tape paper, where they are cured and made solid by polymerization filling pores and cavities. This procedure is used for the insulation of windings in rotating machines, bus bars and condenser bushings [27]. Other applications of epoxy resins include: cable terminations, instrument transformers, switchgear spacers, spacers for gas-insulated substations, bushings, power electronics components packaging and components in circuit breakers [35,36].

The characteristic parameters of epoxy resins depend on the type of primary epoxy compound, the hardener and the accelerator compound used for each specific epoxy compound. Aliphatic amines exhibit higher conductivities due to formation of amine salts or adducts with water. The best dielectric properties are obtained with aromatic anhydride cured resins [27]. Table 4 summarizes the main electrical properties of epoxy resins and factors that influence their magnitudes [26].

Fillers and curing agents also affect the properties of the final cured epoxy compound. Table 5 summarizes the main electrical properties of various amine-cured formulations [26].

In [37], relative permittivity and conductance of bisphenol-A epoxy resin, as a function of frequency and temperature, were characterized using dielectric spectroscopy and it was found that below the glass transition temperature, the above parameters remain almost constant in the frequency range considered (1 × 10^−4^–10 kHz).

Certain characteristics of the epoxy resins can be improved with the addition of nano-sized inorganic fillers [38]. With the addition of nanofillers to epoxy resins, the DC conductivity increases, the permittivity decreases, PD and tracking resistance improve and thermal conductivity and glass transition temperature are increased [39].

The inclusion of nanofillers in epoxy resins increases the resistance to PD activity and enlarge treeing lifetime. In addition, the space charge can be significantly improved. In order to reduce the size of apparatus with gas–solid insulation systems, it is necessary to reduce the electric field strength stress due to the difference in permittivity between dielectric media. Epoxy composites have higher permittivity than neat epoxy resin due to the inclusion of materials such as, alumina (Al_2_O_3_) or silica (SiO_2_), with high permittivity. Permittivity decreases when a high-permittivity filler, such as, titanium dioxide or titania (TiO_2_) is incorporated. However, the improvement in breakdown strength is no so clear because it is more sensitive to spatial distribution, complex interfaces relationships and morphological characteristics associated with ions, dipoles and traps [35].

The addition of SiO_2_ to epoxy resin allows achieving the same low thermal expansion as aluminum or copper conductors. In addition, excellent electrical insulation properties are maintained. However, the viscosity and costs increase [40]. Nanocomposite epoxy resin has higher viscosity than conventional epoxy resin, which affects manufacturability and lifetime [41].

Some of the nanofillers used for dielectric materials include: Barium titanate (BTO), calcium copper titanate (CCTO) [42], silica (SiO_2_) [43], alumina (Al_2_O_3_) [44,45], clay [46], magnesium oxide (MgO) [47] and zinc oxide (ZnO) [28]. Through the analysis of experimental results, it can be concluded that epoxy nanocomposites with clay and Al_2_O_3_ exhibit low values of permittivity and resistivity [48]. However, the AC breakdown voltage is higher with the inclusion of clay than TiO_2_ and Al_2_O_3_ [46]. On the other hand, the addition of SiO_2_, improves the dielectric characteristics of the nanocomposite [49]. In [43], a surface plasma treatment was applied on SiO_2_ nanoparticles (NP) before fabrication of nanocomposites with epoxy resin (bisphenol-A). It was found that the AC breakdown voltage increases 100% for plasma-treated NP-filled nanocomposites with respect to pure epoxy resin and the PD inception voltage magnitude increases in 32.4%. With the inclusion of SiO_2_, the permittivity and tan δ values are slightly affected [50]. In addition, unlike the effect of inclusion of nano-fillers such as TiO_2_ and ZnO, SiO_2_ particles allow to increase the time to breakdown [28].

## 3. PD Modelling Using the Three-Capacitance and Analytical Models

Under high voltage (HV), PD could occur in small gaps or voids inside the resin, resultant from incomplete degassing during the curing process. Different studies about the PD magnitude, rate, gas composition and material degradation have been made in epoxy resins [51,52,53]. It was found that PD characteristics such as PD rate and magnitude are directly dependent on the voltage amplitude. In addition, it was found that the PD magnitude is related to the affected area or void size in the insulation system, however, it cannot be used as the only factor for indicating the insulation life expectancy. The gas content also affects the PD magnitude and rate as well as the material degradation. When oxygen disappears after the early PD stage, nitrogen, moisture and other gases remain that facilitate the appearance of swarming micro PD that cause local erosion, called pits, which indeed result in the initiation of a tree if the electric field strength at the tip of the pit is highly enough.

In [5], three epoxy resins were used for studying the process of void formation and ageing, finding that if a specimen is free of voids, a void can be formed after prolonged electron bombardment in high fields. Once voids are present, they will grow due to deterioration of material produced by high energy electrons accelerated in the cavities. However, it was concluded that voids less than 1 µm in size do not impair the dielectric strength. In [54], it was shown that epoxy surfaces subjected to PD activity suffer chemical and physical modifications, such as the apparition of drops and crystals, and the increase of surface conductivity after few hours of applied voltage.

The PD modelling procedure could be described as follows [55]. First, it is necessary to determine the geometry and electrical characteristics of the object under test and the electric source applied for stablishing the electric field. Second, it is necessary to determine if under the considered conditions, a PD could start. Third, if conditions for a PD are fulfilled, it is necessary to calculate the charge deployed by the streamer discharge and the induced charge in the measurement circuit. Finally, it is necessary to analyse the evolution of charge distribution on cavity surface. The electric field strength inside the cavity is the superposition of the electric field generated by the external source applied to the electrodes, and the electric field strength inside the void created by the surface charge distribution left by previous PD events. The PD models differentiate in the way the electric field inside the cavity is calculated [19].

### 3.1. Stochastic Model for PD Calculations

Through experimentation and analytical studies, it has been found that PD phenomenon is a stochastic process where their properties are describable by time-dependent random variables. The following analysis applies to voids containing electronegative gases, such as air. Essentially, there are two necessary conditions for a PD to start:

- The electric field inside the cavity is greater than the critical value for starting an avalanche (streamer inception)
- A first electron for starting the first avalanche is present (electron generation rate)

These conditions outcome into the stochastic behavior of PD phenomenon determining characteristics such as inception delay, phase occurrence and the number of PD per cycle. The fulfilment of these conditions is affected by factors such as those described in [56]: Probability of first electron injection as a function of electric field strength, ionizing radiation, dynamics of surface charge decay and generation rates of ions and metastables.

#### 3.1.1. Streamer Inception

Streamers are self-sustained discharges controlled by a critical avalanche criterion that defines an inception value given by [57]:

(1) $$E_{inc}={\left(E_{1}/p\right)}_{cr}p\left(1+\frac{B}{{\left(2pa\right)}^{n}}\right)$$

where $E_{inc}$ (V·m^−1^) is the inception electric field strength magnitude, ${\left(E_{1}/p\right)}_{cr}$, *B* and *n* are parameters related to the ionization process in gases and depend on each specific gas, for example, in air the values are, respectively, 24.2 V·Pa^−1^, 8.6 Pa^1/2^·m^1/2^ and 0.5. On the other hand, *p* is the pressure in Pa and *a* is the radius of the void in m. Although Equation (1) was determined in a configuration different to a gas filled void surrounded by a solid dielectric, Callender [16] found that shows good agreement with advanced plasma models and experimental values.

The first condition, related to the threshold value calculated with Equation (1) is merely deterministic and depends on parameters of media, the cavity size and the pressure of gas inside the void.

#### 3.1.2. Electron Generation Rate

Volume and surface emissions are the main mechanisms for the first electron generation rate [55]. Volume generation is related to radiative gas ionization and field detachment of electrons from negative ions. This is the main source of first electrons in voids without previous PD activity. The electron generation rate by volume ionization can be calculated using Equation (2) [23].

(2) $$N_{et}\left(t\right)=C_{rad}\Phi_{rad}{\left(\rho /p\right)}_{0}p\left(\frac{4}{3}\pi a^{3}\right)\left(1-\nu^{-1/n}\right)$$

where $C_{rad}$ is a factor which describes the interaction of the radiation with the gas, $\Phi_{rad}$ (kg^−1^·s^−1^) is the radiative cosmic and radioactive quantum flux density, ${\left(\rho /p\right)}_{0}$ (kg·m^−3^) is the pressure reduced gas density, $\nu =U_{cav}(t)/U_{inc}$, $U_{cav}(t)$ (V) is the voltage across the cavity center and $U_{inc}$ (V) is the inception voltage. After a PD event, there are charges on the surface of void that plays an important role in the second mechanism of electron generation. The electron generation rate due to surface de-trapping obeys the Richardson–Schottky law and can be written as in Equation (3).

(3) $$N_{dt}\left(t\right)=N_{dt0}\exp\left(-\frac{t-t_{PD}}{\tau }\right)\nu_{0}\exp\left(-\frac{\Phi_{dt}-\sqrt{eE_{cav}\left(t\right)/\left(4\pi \varepsilon_{0}\right)}}{kT}\right)$$

where *e* (C) is the elementary charge, $\Phi_{dt}$ (eV) is the effective de-trapping work function, *k* (eV·K^−1^) is the Boltzmann constant, $t-t_{PD}$ (s) is the time elapsed since the latest PD, $\nu_{0}$ (s^−1^) is the fundamental frequency of phonon $E_{cav}$ (V·m^−1^) is the electric field strength inside the void and *T* de temperature in K. $N_{dt0}=\xi \left(q/e\right)$ and ξ is a proportional factor. ξ describes the fraction of charge carriers, which result in the creation of de-trappable electrons and depends on the polarity of charges deployed on the surface and the electric field strength polarity. The initial electron generation is modelled using a random numbers generator, which produces an electron in the time interval [t,t+Δt] with the probability $\left(N_{et}+N_{dt}\right)\Delta t$.

Equations (2) and (3) depend on parameters that are unknown for many materials and conditions, for this reason, other authors have proposed some variations for modelling the stochastic behavior of PD phenomena. In [13], C. Forssén and H. Edin assumed that the generation of free electrons in the void is mainly due to surface emission and the distribution function for a PD is calculated using the Equation (4).

(4) $$F(t)=1-\exp\left(-\int_{0}^{t}N_{e}(t\prime )dt\prime \right)$$

where $N_{e}(t)=N_{e0}\exp\left(\left|U_{cav}(t)/U_{inc}\right|\right)$ (s^−1^) and $N_{e0}$ (s^−1^) is a constant depending on the applied frequency. The instant for a PD is determined comparing the value obtained using the Equation (4) with a random number uniformly distributed between 0 and 1.

Similarly, Illias et al. [58], neglecting the initial electron generation rate from volume ionization, defined the total electron generation rate due to surface emission at the instant *t* as in Equation (5).

(5) $$N_{est}(t)=\left(N_{ed}(t)+N_{ei}\right)\exp\left(\left|U_{cav}(t)/U_{inc}\right|\right)$$

where $N_{ed}(t)=N_{ed0}\left|U_{cav}(t_{PD})/U_{inc}\right|\exp\left(-\left(t-t_{PD}\right)/\tau_{trap}\right)$ (s^−1^), $N_{ei}$ (s^−1^) is a parameter corresponding to the charge de-trapping from polymer loose chain ends, $N_{ed}$ (s^−1^) is the electron generation rate due to charge de-trapping from shallow traps near the cavity surface, $N_{ed0}$ (s^−1^) is a constant depending on the polarity of the electric field in the cavity, $U_{cav}\left(t_{PD}\right)$ (V) is the cavity voltage at the time $t_{PD}$ (s) of the previous PD event and $\tau_{trap}$ (s) is the time constant for charge decay through charge movement into deeper traps. The likelihood of a PD occurrence in the interval [t,t+Δt] is calculated as $L\left(t\right)=\left(N_{et}+N_{dt}\left(t\right)\right)\Delta t$, then is compared with a random number uniformly distributed between 0 and 1.

This stochastic modelling procedure is also used in the three-capacitance model. Considering a uniform distribution of the electric field strength inside the void, the likelihood can be calculated using the voltages in the equivalent circuit for controlling a switch that is in parallel with the void capacitance or the value of the streamer equivalent resistance [59]. More details area presented in Section 3.3.

### 3.2. Analytical Model

The analytical model is also known as induced charge or dipole moment model. This is a field based approach that was initially proposed by Pedersen [60], in which is considered that all the void participates in the PD process, the electric field strength inside the void is uniformly distributed and the charge left by previous PD forms a dipole that induces a charge distribution on the HV electrodes.

The PD starts once there is an electron in the cavity that can be accelerated by the applied electric field and a streamer, a self-sustained discharge, can take place [19]. Due to the high conductivity of the streamer channel, the electric field in the cavity is reduced and below an extinction magnitude, *E_ext_* (V·m^−1^), the streamer development stops. The change in the field within the cavity causes a change in the charge on the electrodes and the charging process is due to the creation of charge carriers as consequence of the ionization process in the gas-filled cavity. After a PD event, the electric charges in the dielectric bulk and voids will induce a proportional charge distribution on the HV electrodes that can be calculated using Equation (6) [61].

(6) $$q\prime =-\int_{\Omega }\lambda \rho_{c}d\Omega -\sum_{j=1}^{N}\int_{S_{j}}\lambda \sigma dS$$

where q′ (C) is the induced PD charge, λ is a proportionality positive scalar function, which is continuous and dimensionless, Ω (m^3^) is the volume of the entire dielectric system, $S_{j}$ (m^2^) is the surface of the *j*-th void, N is the number of voids, $\rho_{c}$ (C/m^3^) is the volume charge density and σ (C/m^2^) is the surface charge density. λ can be determined solving the same electro-geometrical configuration, but considering it as free of charges, so λ satisfies the Laplace’s Equation (7).

(7) ∇·(ε∇λ)=0

**Figure 1:**
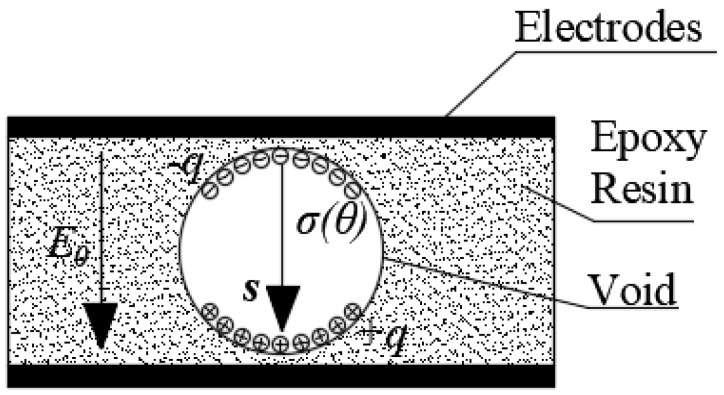
Dipole moment due to charge on cavity surface. *E*_0_ is the electric field strength outside the void due to applied electrodes and **s** is the unit dipole vector.

From Equation (7) λ can be interpreted as the scalar potential distribution in the free charge system per unit of applied voltage and must fulfil the boundary conditions established by (8).

(8) $$\begin{array}{l} \lambda =1\;\mathrm{at}\;\mathrm{HV}\;\mathrm{electrode}\\ \lambda =0\;\mathrm{at}\;\mathrm{grounded}\;\mathrm{electrode}\\ \varepsilon_{r}\frac{\partial \lambda_{DM}}{\partial r}=\frac{\partial \lambda_{VOID}}{\partial r} \end{array}$$

where $\lambda_{DM}$ and $\lambda_{VOID}$ are the solutions at each side of the interface between void, VOID, and dielectric material, DM, and r is the normal direction to the interface. After a PD event, the spatial charge deployed on the void surface produces a field, a Poisson field, that opposes the electrostatic field produced by electrodes in the dielectric material, a Laplacian field [62]. In addition, the total charge in the void must remain zero, giving rise to the appearance of a dipole moment as is shown in Figure 1.

In Figure 1, **s** is the vector pointing from the negative to positive polarity charges. The dipole moment can be expressed as in Equation (9).

(9) $$p=\int_{\Omega }\rho_{c}rd\Omega +\int_{S}\sigma rdS$$

where **p** (C·m) is the dipole moment of the charges on the void surface and **r** is the position vector of the element of charge. Considering that the void is smaller than the dielectric bulk, the gradient of λ inside the void is uniform and the induced charge due to the dipole in the measurement circuit is given by the Equation (10).

(10) q′=−p·∇λ

In Equation (10), ∇λ can be calculated as $\nabla \lambda =K\nabla \lambda_{0}$, where $\lambda_{0}$ is the response function at the void location for the condition of dielectric system without voids and free of electric charges, and *K* is a void shape factor that depends on the geometry of the void. For ellipsoidal voids Equation (10) can be rewritten as in Equation (11) [57].

(11) $$q\prime =-K\Omega \varepsilon \left(E_{inc}-E_{ext}\right)\cdot \nabla \lambda_{0}$$

where $E_{inc}$ (V·m^−1^) is the inception value of electric field strength, ε (F·m^−1^) is the dielectric permittivity of media and $E_{ext}$ (V·m^−1^) is the electric field strength below, which there is not ionization and the streamer development ceases. Because the cavity is small in comparison with the dielectric bulk size, it could be assumed that the electric field strength inside the cavity is uniform.

PD events leave electric charges on the surface of voids that are redistributed and neutralized by surface currents. For that reason, the electric field strength inside cavities has a complex dependence on time because it varies depending on time variations of external applied electric field and the decay of electric charges on the void as a function of time.

**Figure 2:**
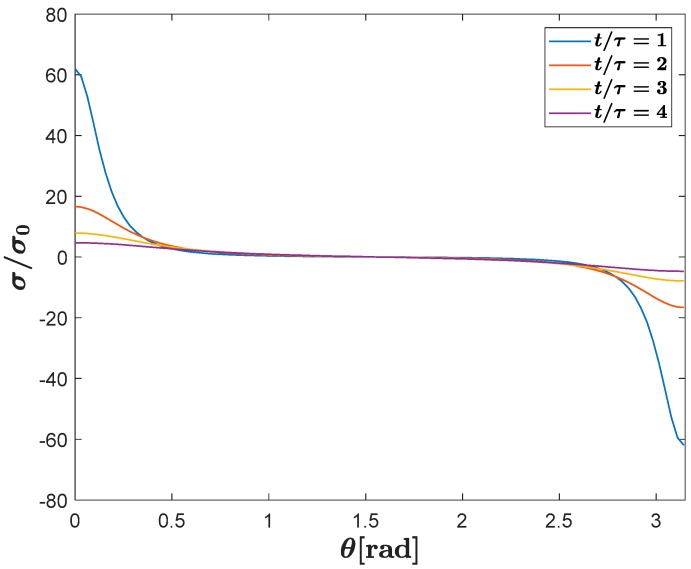
Surface charge distribution on the void surface as a function of time for 0 ≤ *θ* ≤ *π*. *σ*_0_ = *q/4πa^2^* and *τ = ε*_0_*a/k_s_*. It is assumed *q* = 80 pC, *k_s_* = 1 × 10^−12^ S·m^−1^ and *a* = 0.55 mm.

Figure 2 shows the behavior of surface charge density distribution on the cavity surface as a function of time for a spherical cavity immersed in an epoxy resin with $\varepsilon_{r}=4.4$.

In Figure 2 $\sigma_{0}=q/4\pi a^{2}$ (C·m^−2^) and $\tau =\varepsilon_{0}a/k_{s}$ (s) where $k_{s}$ (S·m^−1^) is the cavity surface conductivity. As can be seen, the density of charge is symmetrically distributed on the cavity surface around the axis of symmetry and has a time constant that is mainly dependent on the relative permittivity [63].

In the analytical approach, the surface charge dynamics is modelled using the Ohm’s law [23]:

(12) $$-\frac{dq}{dt}=\left(\frac{\pi }{2}\right)k_{s}E_{cav}2a$$

Illias et al. [58] through an analysis of mobility of charges on the surface and experimental results, proposed modelling the surface charge decay using a cavity surface conductivity as a function of electric field. In this model, surface conductivity is dynamically changed depending on the polarity of the electric field inside the cavity respect to electric field produced by surface charge density. The time decay is considered as an equivalent RC time constant $\tau_{s}$ that can be expressed as [64]:

(13) $$\tau_{s}=\frac{\varepsilon_{0}a}{2k_{s}}$$

Considering that the Poisson field is produced by the charge in the system distributed in the entire volume and deployed on the void surface, this equation has to be considered to estimate the PD charge. Lemke [65] used the dipole moment approach and an energy balance analysis for expressing the induced charge as in Equation (14).

(14) $$q\prime =p\cdot \frac{E_{inc}}{U_{inc}}$$

Considering PD as streamer discharges and assuming that the Laplacian field remains constant, because the PD process is in the order of nanoseconds [66], the dipole moment can be assessed using the following semi-empirical expression:

(15) $$\left|P\right|=\left(270\mathrm{pC}/\mathrm{mm}\right)d_{c}^{2}$$

where $d_{c}$ (mm) is the void diameter. Lemke used this expression for analyzing the PD charge transfer in polymeric power cables. This expression is applicable for virgin voids, without previous PD, and 0.1 mm < $d_{c}$ < 2 mm.

A generalized approach to PD modelling was presented by Niemeyer [55], where the analytical model is used including the effects of charge decay and memory, and simulation results for different voids and protrusions were presented. In the same way, in [23], a stochastic discharge model based on the analytical approach is presented and some comparisons with measurements for different periods of time of the applied voltage showed good agreement. This analytical approach has been used for determining the theoretical magnitude of PD charges in analysis of defects in solid dielectric cables [67]. Through a mathematical procedure, the minimum theoretical discharge initiation voltage can be determined, however, as PD will not initiate at this voltage, this value should be increased in order to promote the PD appearance during laboratory tests. In [68], the analytical approach is used for determining the discharge characteristics at DC voltage and it was found that for this case the electrical conductivity plays a fundamental role in the inception voltage, repetition rate and discharge magnitudes, however there is not experimental validation.

In this model, it is considered that the electric field strength inside the cavity is uniform, the electric field strength in the bulk of dielectric material remains unaltered during the PD event and the entire cavity surface is affected by the PD event. In [63], through the analytical solution of field equations, it was shown that the electric field strength inside cavity after a PD, is not uniform, its distribution depends on the surface charge decay process. Additionally, the electric field outside the cavity is affected by the charge distribution on the cavity surface [69,70]. However, in this model, the electric field calculations are considered in the center of the void, where the electric field is almost uniform.

#### Other Analytical Models

Other analytical models have been proposed [71,72], where analytical expressions are used. However, the deterministic formulation is in contrast with the stochastic behavior of PD phenomena that is deduced from theoretical and experimental studies. On the other hand, other analytical models have been presented where the PD process is modelled using mathematical relationships between electric and geometric variables and the result is the probability density for a PD occurrence [73]. The PD phenomenon is treated as a stochastic process consisting of short-duration discharges and charge carrier drift, and recombination intervals between the discharges. In this approach, no simulations are required because, the calculations involve few basic physical parameters and a master equation in which the dynamics of the internal field forms a piecewise deterministic Markov process. This is an analytical approach with good agreement with experimental results. However, it does not consider all the physical elements involved, such as memory effect and charge decay, because this dependency is reduced to a single decay time constant. On the other hand, the inception and residual values of electric field inside the cavity remains constant during the simulation process, disregarding changes in temperature and pressure.

### 3.3. Three-Capacitance Model

**Figure 3:**
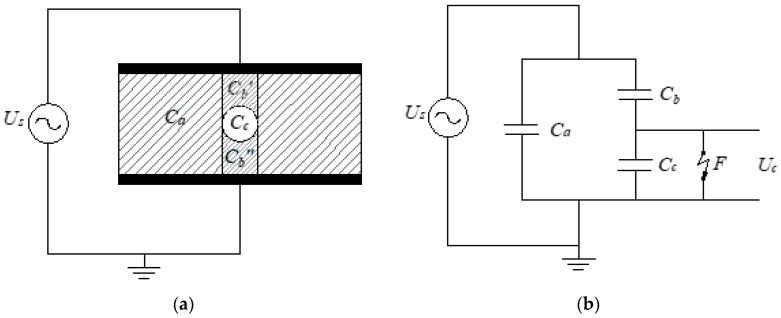
Whitehead three-capacitance model: (**a**) bulk of dielectric material with a spherical void and capacitances of components in series and parallel with the void; (**b**) “abc” equivalent circuit, F is the spark-gap representing the PD event.

The three-capacitance model was initially proposed by Gemant and Philippoff in 1932 for estimating the power losses in power cables due to discharges in voids [74]. They made experimental measurements and found that the PD rate was in good agreement with theoretical calculations using the equivalent circuit [61]. In 1951, Whitehead proposed the modified equivalent circuit shown in Figure 3, usually known as the “abc” model [75]. This circuit allows calculating a numerical relationship between the charge in the measurement circuit and the internal charge in the cavity.

In Figure 3, $C_{a}$ (F) is the capacitance of the bulk of dielectric material, $C_{b}$ (F) is the equivalent capacitance of dielectric column in series with the void, resultant from $C_{b}\prime ||C_{b}\prime \prime$, and $C_{c}$ (F) is the capacitance of the void which is short-circuited by the spark gap F.

A PD is simulated with the closing of the spark gap F. F will be closed when the voltage across the void capacitance $U_{c}\left(V\right)$ is equal to or higher than the inception voltage $U_{inc}\left(V\right)$ until $U_{c}\left(V\right)$ is equal to or lower than the extinction voltage $U_{ext}\left(V\right).$ The voltages controlling the changes on the spark gap F can be calculated using the inception and residual fields and considering the geometry of the void [55].

**Figure 4:**
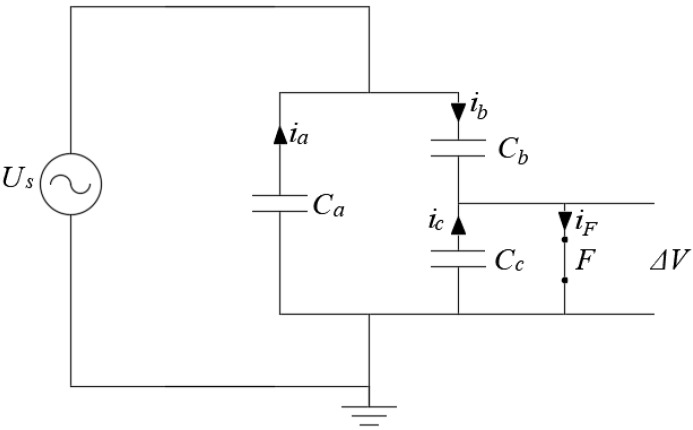
Three-capacitance model representation during a PD event. When a PD occurs, Cc is short-circuited by the spark gap *F* and a transient current will flow. In addition, voltages will be modified by the transient phenomenon.

When a PD occurs, $C_{c}$ is short-circuited thought the spark gap F and a transient current will flow due to the change in voltage as it is shown in Figure 4, where $i_{F}=i_{c}+i_{b}\;(A).$ The real PD charge, q (C), in the void can be calculated using the Equation (16).

(16) $$q=\Delta U\cdot \left(C_{c}+\frac{C_{a}\cdot C_{b}}{C_{a}+C_{b}}\right)\;$$

where ΔU (V) is the voltage change across the void produced by the PD. From Figure 4, it is clear that during the PD event, the current associated to ΔU will flow through $C_{b}$ and $C_{a}$. The change in the charge on the electrode, the induced charge, can be calculated as in Equation (17).

(17) $$q\prime =\Delta U\cdot C_{b}\;$$

As can be seen from Equations (16) and (17), the real charge in the void and the charge in the measurement circuit are different [11]. The above relationships assume that the applied test voltage induces charges on the cavity surface, which means that the internal charge has already been produced just before a PD event is ignited. This is a violation to the causality law [61].

For modelling PD using the three-capacitance method, different efforts and applications have been made, which can be classified into the following categories:

- Spark gap simulated using a switch
- Variable gap resistance
- Variable capacitance

#### 3.3.1. Spark Gap Simulated Using a Switch

**Figure 5:**
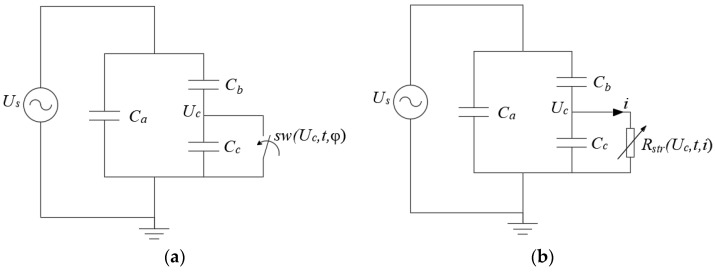
PD three capacitance equivalent circuit: (**a**) PD simulation using a switch in parallel to *C_c_*. The switch is controlled using voltage, phase and time as variables; (**b**) PD simulation using a variable resistance in parallel to *C_c_*. The value of the resistance is controlled using voltage, current and time as variables.

In this category, the streamer propagation process is simulated using a switch, which is in parallel to $C_{c}$, as it is shown in Figure 5a).

The switch operation can be controlled using logical operations when the voltage across the void exceeds the threshold value or, when experimental q-φ data are available, for specific instants. In addition, the switch closure can be controlled taking into account the stochastic behavior of the PD phenomena. In [76], this three capacitance model was used for determining the theoretical PD distribution using a Monte Carlo simulation taking into account experimental results. They found that the discharge area, the oxygen content and the pressure of gas in void affect the extinction voltage, which indeed, affects the PD inception time lag. In a similar way, in [77], an integral equation was proposed for describing the stochastic fluctuations of PD occurrence in the three capacitance model when the gap closure have stochastic fluctuations in the time delay under ac voltage. This same approach was used for explaining the PD characteristics obtained in the IEC(b) [78] electrode system [79].

On the other hand, the three-capacitance model can be used for simulating PD under DC and higher frequency voltages. In [80], a modified three capacitance model for describing the PD behavior for DC voltage was developed where resistances for considering conduction processes were added. When comparing simulated results with measurements, some differences were found that were related to the assumed discharge area and voltage drop. The distribution functions of the discharge magnitude and the time between two discharges was studied through simulations. It was found that the extinction and ignition voltages were similar and the PD rate is dependent on the test voltage and its ripple content. A similar modified three-capacitance model with resistances was used in [81] for studying the effect of applying higher frequency voltages on the PD behavior in cavities with different size and location in the dielectric. They found that the PD rate decreases at higher frequency, although the PD magnitude increases.

In [82], PD pulse waveforms from the three-capacitance model were obtained and studied on different supply voltages and different void sizes. Time–Frequency (TF) analysis was performed by applying Short-Time Fourier Transform (STFT) and different characteristics such as amplitude, occurrence, frequency distribution, intensity of the frequencies, were analyzed.

In [83], the three-capacitance model was modified including a resistance for modelling the surface conduction process and the insulation resistance variation with aging. Also, an empirical expression for taking into account that the PD magnitude increases when temperature is elevated is included [84].

A three-capacitance model implemented in Simulink for a cylindrical void was used in [85] to make a characterization of the PD pulses on duration, magnitude and frequency content for different geometries. A similar study, but also including the measurement circuit was reported in [86], the effect of the void axis orientation respect to the applied electric field on the PD magnitude was analyzed. It was found that the PD magnitude is higher when the void axis is perpendicular to the applied electric field.

In [87], a modified three-capacitance model was implemented in Simulink which includes and additional capacitor in series with the gap whose operation is controlled externally using values taken from experimentally measured q-φ diagrams. The additional capacitor allows considering the additional electric field inside the void generated by previous PD. However, the discharge occurred once because of the non-repetitive switching mode of the breaker. A similar study, but implemented in EMTP-RV was reported in [88] where different void sizes and types of dielectric materials were considered and an additional resistor and a capacitor were included in the three capacitance model for taking into account the additional electric field generated by the electric charge of previous PD.

#### 3.3.2. Variable Gap Resistance

The streamer propagation can be modelled using a variable gap resistance approach. The circuit in Figure 5b) is a modified version of the three capacitance model of Whitehead [75]. The value of the resistance is dynamically modified taking into account the voltage threshold and the solution of the electric circuit allows considering some of the physics related to PD phenomena: stochastic behavior, thermal and pressure effects, charge decay, streamer propagation, extinction voltage, etc.

In [89], the nonlinearity of the PD phenomena is considered using a voltage-and time-dependent resistance. The proposed model considers the measuring circuit and the voltage associated to surface charge on the void through a mathematical function controlling the value of the resistance of streamer ($R_{str}$) after a PD event, which allows the voltage across the capacitance of the void to build up after the PD event. The mathematical function considered is the decay exponential function given by the Equation (18).

(18) $$R_{str}=R_{0}\cdot \exp\left(-t/\tau_{R}\right)$$

where $\tau_{R}\;(s)$ is the time constant of the simulated discharge and $R_{0}\;(\Omega )$ is the initial insulation resistance of the gap.

In [59], a similar approach as previously described is used. The PD current path is represented using a voltage and current-dependent streamer resistance, which allows maintaining its low resistance state during the avalanche due to the current dependence. Additional resistances for considering the conduction around the cavity and the effect of the conductivity of the bulk of dielectric material were added. The functional form for $R_{str}$ (Ω) corresponds to Equation (19).

(19) $$R_{str}=R_{0}\cdot \exp\left(-\left|U_{c}/U_{inc}\right|-\left|i/I_{0}\right|\right)\;$$

where $U_{inc}\;(V)$ is the inception voltage, $I_{0}\;(A)$ is the critical current for an avalanche. A comparative analysis with measured data showed good agreement with simulation results.

In [90], it was presented an approach for modelling PD in spherical voids in epoxy resin which is based on a time varying conductance of the void considering multi-stresses conditions (voltage, temperature and pressure). The PD phenomena is modelled with a variable conductance of plasma, G(t) (S), considering only direct ionization and recombination as shown in Equation (20).

(20) $$\frac{dG\left(t\right)}{dt}=K_{prod}W\left(U_{i}\right)G\left(t\right)-K_{2}^{rec}G{\left(t\right)}^{2}-K_{1}^{rec}G\left(t\right)$$

where $K_{prod}{\;(s}^{-1})$ is the ionization coefficient and $K_{n}^{rec}{\;(s}^{-1})$ are the recombination coefficients, n=2 for higher conductance values. The trigger function $W\left(U_{i}\right)$ is used for checking the fulfilment of the required conditions for a PD event and takes into account the stochastic behavior of the PD phenomena through a Weibull probabilistic function. Simulation results exhibit good agreement with experimental data found in the literature [91]. This model was modified with the inclusion of surface and volume resistances of the bulk of dielectric material for modelling the PD behavior under a voltage stress close to the DC waveform [92]. The simulation results show that the PD rate decreases when the sample is subjected to a voltage stress close to the DC waveform, which could depend on the space charge accumulation phenomenon.

#### 3.3.3. Variable Gap Capacitance

In order to conciliate the three-capacitance method assumptions with real PD physics, a method of variable capacitance was proposed in [93]. In this study, a detailed analysis of the critics to the three-capacitance method was made. It was concluded that the capacitive model is able to represent the transient current in electrodes caused by the space charge and the PD charge, which is linked to the partial capacitances and new potentials of the electrodes. The total capacitance of the system is expressed as in Equation (21).

(21) $$C\prime \prime =C\prime \;(F)+\left(\frac{K\Omega \varepsilon \left(E_{inc}-E_{res}\right)}{U-\Delta U}\cdot \nabla \lambda_{0}\right)$$

where C′ (F) is the capacitance of the capacitor with the insulation immersed between the plates. Terms in the second parenthesis are defined as in [57], K is a dimensionless shape factor, Ω  (m^3^) is the volume of the void, ε  (F·m^−1^) is the permittivity of the dielectric media, $E_{inc}$ (V·m^−1^) and $E_{ext}$ (V·m^−1^) are, respectively, the inception and extinction electric field strength magnitudes, $\lambda_{0}$ is the electric scalar potential per unit of applied voltage and U−ΔU (V) is the voltage after the PD event. The proposed model was implemented in Matlab. However, any results or comparisons were presented.

#### 3.3.4. Transients in Measured Variables and Critics to Capacitive Model

After a PD event, the potential on the electrode drops by ΔU but the charge on the electrode increases by ΔQ, due to charge supplied to the electrode from the external system. PD can be detected in measurement circuits as transient variations in voltages and currents, so it is necessary to relate those variables with the charge on the void surface, which is the main variable of interest. Before a PD event, the potential and the charge at the HV electrode are, respectively, U and Q. After a PD event, the potential and charge are U−ΔU and Q+ΔQ, where ΔQ is the charge transferred to electrodes from the applied HV source. Using the Green’s reciprocal theorem and the definition of capacitance in electrical circuits [94], the induced charge can be expressed as follows:

(22) q′=CΔU+ΔQ≈CΔU

where C (F) is the capacitance of the system. The impedance of the circuit is large enough for the current related to a PD event, so ΔQ (C) can be disregarded in comparison to CΔU. Taking into account that the capacitance of the systems does not change during the PD event, transients cannot be related to a change in the capacitance of the system. On the other hand, during the PD process, the transient current related to the streamer must be equal to the displacement current in the dielectric material in series with the void. This means that the charge detected at the electrodes in the measurement circuit must be equal to the internal PD charge, which is in contrast to the apparent charge concept presented in the capacitive model [61].

In the three-capacitance model, the surface charge dynamics can be modelled using the variable gap resistance approach because the magnitude of the resistance can be numerically controlled taking into account the polarity of the applied voltage and the time elapsed after the latest PD event, similar to the RC time constant approach.

Table 6 exhibits a summary of the main parameters required for implementing simulations in three capacitance and analytical models, their main advantages and disadvantages are also included.

Models can be used for analyzing media where parameters listed in Table 6, are known. However, it must be considered that parameters in the stochastic model must be also known for the same media under analysis. Parameters of stochastic model listed in Table 6, and related to Equations (1)–(3), correspond to epoxy resins [23]. The model can also be applied to other materials if the stochastic model is modified using experimental measurements [18,59].

## 4. Case Study

**Figure 6:**
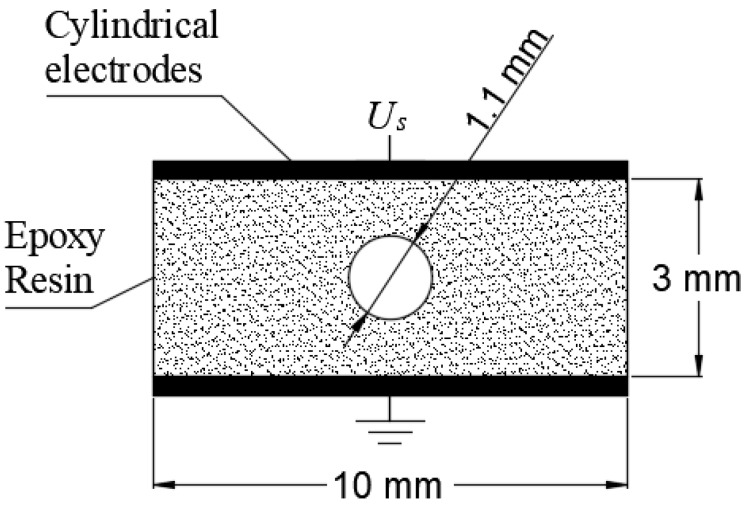
Geometry of the case study. A spherical void with 1.1 mm in diameter immersed in an epoxy-resin dielectric bulk between two parallel plates.

A case study is simulated using the analytical and three-capacitance models and the results are compared with measured values in order to analyze their performance and capabilities. Figure 6, shows the geometry of the case study. It corresponds to a linear, homogenous and isotropic dielectric bulk of epoxy resin (bisphenol-A) of 3 mm thickness and 10 mm diameter between 2-parallel plates. A spherical void, filled with air, with a 1.1 mm diameter is included in the center of the geometrical shaping. An 18 kV, 50 Hz sinusoidal voltage source ($U_{s}$) is applied to upper electrode while the lower is grounded. This same configuration was used by Illias et al. [15] for comparing the performance of analytical and FEA models for PD analysis.

Table 7, summarizes the parameters considered during the simulation process. In order to analyze the applicability and the performance of analytical and three capacitance model, comparisons with measured values reported in [15] are made, and for this reason, the same stochastic approach used by the authors of the that article, which is similar to Equation (5), is used in the present study.

The value of the initial electron generation rate due to surface emission, $N_{es0}$, depends on the polarity changes of the cavity voltage between two consecutive PD. Parameters defined in Table 7, also correspond to parameters required in the analytical model.

### Three-Capacitance Model Parameters Definition

**Figure 7:**
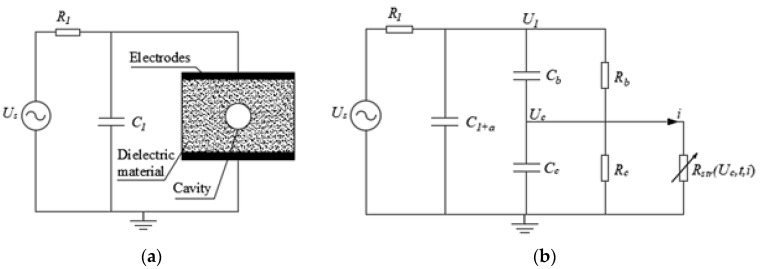
Equivalent circuit for modelling PD considering the variable resistance of gap: (**a**) Epoxy resin dielectric bulk with the measurement circuit; (**b**) three-capacitance equivalent circuit including the variable resistance.

In this study, a modified version of the equivalent circuit presented in [59] is implemented for considering the same stochastic approach as in the analytical model. Figure 7 shows the dielectric bulk with the HV source applied (a), and the equivalent circuit implemented for PD simulating (b).

In Figure 7, $R_{1}$ and $C_{1}$ are, respectively, the series impedance and coupling capacitor in the measurement circuit. $C_{1+a}$ is the equivalent capacitance corresponding to $C_{a}+C_{1}$, $R_{b}$ represents the conductivity of the bulk of dielectric material, $R_{c}$ represents the surface and bulk conduction around the cavity. $R_{str}\left(U_{c},t,i\right)$ is the resistance of the PD streamer and is a function of voltage and current of the streamer, Equation (19). Many mathematical functions, simulating the discharge behaviour in the cavity, have been studied and the optimum representation proved to be a decay exponential function as Equation (19) [95]. In addition, this expression allows considering the variation in the resistance of the streamer due to conduction heating. $R_{0}$ is normally chosen much greater than $R_{c}$ and its value allows to control the numerical convergence.

Using the analogy principle, from Equation (17), the apparent PD charge in the three-capacitance model can be calculated as $q\prime =\Delta U\cdot C_{b}\;(C)$, while from Equation (11) the apparent PD charge in the analytical model can be calculated as q′=ΔU·g (C), where ΔU is the change in voltage through the void due to a PD event. As can be seen g, is a constant that is equivalent to the capacitance of the dielectric material in series with the void. On the other hand, the other capacitances in the circuit can be calculated using the capacitance definition applicable to electric circuits given by Equation (23) [94].

(23) $$C=\frac{\int_{vol}\varepsilon E^{2}dvol}{U^{2}}$$

Table 8, shows the parameters of the electric circuit for this study.

A Matlab code was implemented for simulating each model. The differential equations for the circuit in Figure 7, were solved using the Matlab function ode45, which allows the solution of nonstiff differential equations using the medium order method.

## 5. Results and Discussion

The case study was simulated using the analytical and three-capacitance model for 500 periods of the AC 50 Hz, 18 kV voltage wave. The time step when there is not feasibility of PD is defined as 4.0 × 10^−5^ s, and during PD, as 1 ns. Simulation results are summarized in Table 9, in addition, measured values for the same case study reported in [15] are presented in order to make comparisons.

As can be seen from Table 9, the results obtained using the analytical model are in reasonable agreement with measured ones, presenting the higher difference, 13.767%, for the maximum PD magnitude. On the other hand, comparing with measured values, the three-capacitance model, presents less accurate results, with the higher difference, 20.381%, for the maximum PD magnitude. As expected, the three-capacitance model is less accurate than the analytical model. However, the calculated values are of the same order when compared with measured values and allows an approximated quantitative and qualitative indication of the phenomenon.

Simulations were made using eight processors (CPUs) Intel Xeon E5-2670. The simulation using the analytical model took 270 s, while the three-capacitance model took 2780 s.

**Figure 8:**
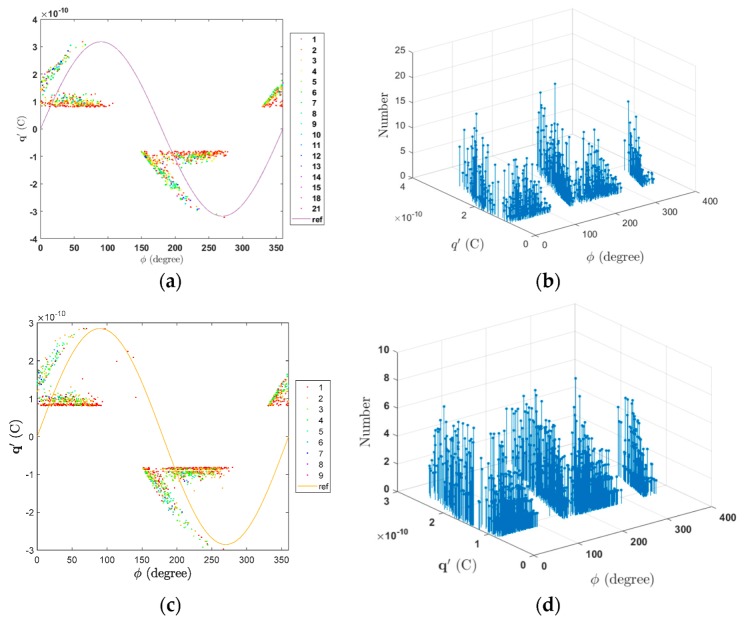
q-φ-n diagrams for the case study: (**a**) 2D representation of the q-φ-n diagram using the analytical model; (**b**) 3D representation of the q-φ-n diagram using the analytical model; (**c**) 2D representation of the q-φ-n diagram using the three capacitance model; (**d**) 3D representation of the q-φ-n diagram using the three capacitance model.

Figure 8 presents the q-φ-n diagrams for the case study using the analytical and the three-capacitance models.

The q-φ-n diagrams in Figure 8 exhibit a structure in good agreement with the measured one [15], are composed by a horizontal bar distribution close to the minimum PD charge magnitude plus a rabbit-ear like distribution where higher PD charge magnitudes occur. However, in Figure 8c,d, it can be seen that the PD rate is different in comparison to the analytical model results, with a lower concentration of PD on the horizontal bar. The rabbit-ear-like distribution is due to PD events after changes in polarity of $U_{cav}$ and the three-capacitance model is able to reproduce this behavior, however, the charge decay and memory effects are not well modelled using fixed values resistances as $R_{c}$ and $R_{b}$ in Figure 7. The resistance of the series insulation affects not only the PRPD pattern structure, also affects the PD rate because it controls the magnitude of the applied voltage to the cavity, represented by $C_{c}$ in the equivalent circuit of Figure 7. On the other hand, the equivalent resistance to conduction around the cavity, $R_{c}$, controls the charge decay effect on the void of surface. These two parameters are very important for the good performance of the model, however sometimes are determined through empirical procedures [83]. A good approximation may be using an adaptive fitting procedure for adjusting the values taking in to account the measured PRPD pattern. Simulations were carried out using a MATLAB application implemented by authors named PDSym1S developed for these purposes [12].

## 6. Conclusions

A brief summary about epoxy resins characteristics, properties and applications as insulating materials have been made. The theoretical background and state-of-the-art of analytical and three-capacitance models are presented. The analytical and three-capacitance models were implemented in a computer algorithm and a case study was simulated. Results of both models show good agreement with experimental values. The three-capacitance model presents greater differences compared to measured values.

The modified three-capacitance model implemented here allows considering the same stochastic approach used in analytical and FEA models and the charge decay process due to conduction around the cavity and through the dielectric bulk. On the other hand, the functional expressions for streamer resistance permit considering the variation in the resistance of the streamer due to conduction heating. However, the strong non-linearity included in the equivalent circuit by the streamer resistance generates numerical difficulties and some variables, such as, the initial resistance of the streamer, $R_{0}$ and the series resistance of the insulation, $R_{b}$, must be adjusted to avoid numerical errors. In addition, the stiffness of the problem makes it necessary to use an implicit time step method with minor steps, which makes the simulations computationally demanding.

Although the three-capacitance model is not as accurate as the analytical one, it can be used as a qualitative tool in teaching environments and as a complement of condition monitoring tools, for example, in locating PD in power transformers using electrical measurements.

Extinction field magnitude as the PD stop criterion must be revised according to deductions made by Callender [22]. Multhypisical analysis must be implemented under ageing conditions when pressure in the cavity, which affects the stochastic model, is a complex function temperature and applied voltage [96]. Simulation results at different ageing conditions must be correlated with patterns used in diagnosis analysis. Future work must consider physical and chemical interactions on the void surface through the cavity surface conductivity [97].

The curing process plays a fundamental role in the achievement of ideal epoxy resin characteristics. Vitrification and gelation processes must be considered when modelling epoxy resins. Future work must consider the PD behavior in epoxy resins under both conditions.

## Figures and Tables

**Table 1:** Epoxy resins for industrial applications [18].

Epoxy Resin	Characteristics
Bisphenol-A	The curing process can occur at room temperature with the addition of triethylene tetramine.
Cycloaliphatic epoxy	Has fully saturated molecular structure, which contributes to a good Ultra Violet (UV) stability, good thermal stability and excellent electrical properties.
Trifunctional	The cured epoxy resin exhibits excellent chemical resistance, good UV blocking effect and good thermal stability.
Novolac	Excellent thermal, chemical, and solvent-resistance properties due to their high cross-linking densities.
Biobased	Low cost and biodegradability.
Fluorine-containing	High-chemical resistance, low coefficient of friction, low dielectric constant, low water absorption, and broad use temperature.
Phosphorus-containing	Flame-retardant, they produce less toxic gas and smoke than halogen-containing compounds.
Silicon-containing	Environmentally friendly flame retardant

**Table 2:** Characteristics of amine hardeners used in low-molecular-weight bisphenol A-based epoxy resins, adapted from [3].

Hardener	Parts Used per 100 Parts Resin	Typical Curing Temperature	Maximal Heat Distortion Temperature of Cured Resin (°C)	Applications
Diethylenetriamine (DETA)	10–11	Room-Temperature	110	General purpose
Diethylaminoropylamine (DEAPA)	7	Room-Temperature	97	General purpose
Meta phenylene di amine (MPDA)	14–15	150 °C (4–6 h)	150	Laminates
4,4′-diaminodiphenylmethane (DADPM)	28.5	165 °C (4–6 h)	160	Laminates
4,4-Diamino diphenyl sulphone (DADPS)	30	160 °C (8 h)	175	Laminates
Piperidine	5–7	100 °C (3 h)	75	General purpose
Triethylamine	10	Room-Temperature	-	Adhesives
Benzylideneacetone (BDA)	15	Room-Temperature	-	Adhesives
Tri(dimethtylaminomethyl)phenol (TDMAMP)	6	Room-Temperature	64	Adhesives, coatings
2-Ethyl hexoate salt of TDMAMP	10–14	-	-	Encapsulation

**Table 3:** Characteristics of anhydride hardeners used in low-molecular-weight bisphenol A-based epoxy resins, adapted from [3].

Hardener	Parts Used per 100 Parts Resin	Typical Curing Temperature	Maximal Heat Distortion Temperature of Cured Resin (°C)	Applications
Phtalic	35–45	120 °C (24 h	110	Casting
Hexahydrophtalic	80	120 °C (24 h)	130	Casting
Pyromellitic	26	220 °C (20 h)	290	High temperature
Nadic methyl	80	120 °C (16 h)	202	High temperature
Dodecenylsuccinic	80	100 °C (2 h) + 150 °C (2 h)	38	Flexibilizing
Chlorendic	100	180 °C (24 h)	180	Flame retarding

**Table 4:** Electrical properties of epoxy resins (information adapted from [26]).

Property	Value	Factors Affecting Its Magnitude
Permittivity	3–6	Frequency (Higher frequencies cause slight increase), Temperature (Higher temperatures cause increase), Fillers.
tan δ	0.003–0.03	Frequency (Higher frequencies cause slight increase), Temperature (Higher temperatures cause increase), Fillers.
Conductivity	10^−10^–10^−13^ S·m^−1^	Temperature (Higher temperatures cause sharply increases), moisture content and humidity exposure.

**Table 5:** Electrical properties of cured epoxy resin (information adapted from [26]).

Property	Value
Permittivity at 1 kHz and 27 °C	4.25–6.35
tan δ at 1 kHz and 27 °C	0.005–0.30
Conductivity at 27 °C	1.67 × 10^−12^–1.25 × 10^−9^ S·m^−1^

**Table 6:** Summary of main characteristics of analytical and three-capacitance model.

Model	Parameters for Implementing Simulations	Advantages	Disadvantages
Three Capacitance	Permittivity of media, void surface conductivity, volume resistivity.	The current pulse through the electrodes can be directly calculated. The PD phenomena is analyzed though voltages relationships.	It is difficult to implement due to non-linearities in the circuit. It is assumed that void surface is equipotential and that the internal charge has already been produced just before a PD event is ignited.
Analytical	Permittivity of media, void surface conductivity.	Computationally efficient. Reliable results due field approach. Multiphysical analysis can be easily implemented for different geometries and electrodes configurations due to field analysis.	The current pulse cannot be directly calculated. The surface charge density is uniformly distributed. PD affect all the cavity volume. It is considered that the electric field strength inside the cavity is uniform.

**Table 7:** Parameters definition for the simulation of the case study.

Parameter	Description	Value
$\varepsilon_{mat}$	Material relative permittivity	4.4
$\varepsilon_{cav}$	Cavity relative permittivity	1
$\sigma_{mat}$	Material conductivity	1 × 10^−13^ S·m^−1^
$\sigma_{scav}$	Cavity surface conductivity	1 × 10^−12^ S·m^−1^
$E_{inc}$	Electric field strength magnitude for streamer inception	3.40 × 10^6^ V·m^−1^
$E_{ext}$	Electric field strength magnitude for streamer extinction	0.44 × 10^6^ V·m^−1^
$N_{es0}$	Initial electron generation rate due to surface emission	10,000/5000 s^−1^
$N_{ev}$	Initial electron generation rate due to volume ionization	10 s^−1^
$\tau_{dec}$	Charge decay time constant	2 ms

**Table 8:** Parameter definition for the three-capacitance model.

Parameter	Definition	Value
$R_{0}$	Initial resistance of streamer	1 × 10^16^ Ω
$I_{0}$	Critical current for avalanche	0.01 nA
$R_{c}$	Equivalent resistance to conduction around the cavity	6.37 × 10^11^ Ω
$C_{c}$	Capacitance of the cavity	2.69 × 10^−14^ F
$R_{b}$	Series resistance of the insulation	1 × 10^13^ Ω
$C_{b}$	Series capacitance of the insulation	2.47 × 10^−14^ F
$R_{1}$	Series resistance of measurement circuit	1 × 10^5^ Ω
$C_{a}$	Capacitance of the homogenous part of the insulation	1.01 × 10^−12^ F
$U_{inc}$	Cavity voltage magnitude for streamer inception	3.740 kV
$U_{ext}$	Cavity voltage magnitude for streamer extinction	0.484 kV

**Table 9:** Simulation results summary and measured values for the case study.

Method/Variable	Measured ^1^	Analytical		Three-Capacitance	
		Magnitude	Error (%)	Magnitude	Error (%)
PD per cycle	6.5	6.490	0.154	6.134	5.631
Minimum PD magnitude, *q_min_* (pC)	80	80.302	0.378	81.318	1.648
Maximum PD magnitude, *q_max_* (pC)	373	321.650	13.767	296.980	20.381
Mean PD magnitude, *q_mean_* (pC)	101	104.100	3.069	101.610	0.604

^1^ Measured values reported in [15].
